# Early breastfeeding as protective factor for preschool emotional and behavioral health

**DOI:** 10.1007/s00431-025-06666-9

**Published:** 2025-12-24

**Authors:** Susana Vargas-Pérez, Carmen Hernández-Martínez, Núria Voltas, Victoria Arija, Josefa Canals-Sans

**Affiliations:** 1 Research Group in Nutrition and Mental Health (NUTRISAM), Rovira i Virgili University, Tarragona, Spain; 2 Research Center for Behavioral Assessment (CRAMC), Rovira i Virgili University, Tarragona, Spain; 3 Pere Virgili Institute for Health Research (IISPV), Rovira i Virgili University, Reus, Spain

**Keywords:** Breastfeeding, Behavioral problems, Emotional problems, Child development, Child mental health

## Abstract

To examine the association between any breastfeeding duration and emotional and behavioral problems in 4-year-old children, considering psychosocial, demographic, and perinatal variables. The sample included 564 children. Any breastfeeding duration was categorized into four groups: no breastfeeding, 1–4 months, 4–8 months, and more than 8 months. Emotional and behavioral development was assessed using the Child Behavior Checklist 1^½^–5 (CBCL 1^½^–5). Multiple linear and logistic regression models were applied to explore associations between breastfeeding groups and CBCL 1^½^–5 outcomes. Any breastfeeding for 1–4 months was associated with lower scores on internalizing (*β* = − 4.21; *p* = 0.014) and externalizing (*β* = − 3.30; *p* = 0.044) problems scales, including emotional reactivity, anxiety/depression, somatic complaints, withdrawn behavior, and aggressiveness. It also reduced the risk of clinical scores for internalizing problems (OR = 0.035; *p* = 0.010) and symptoms compatible with autism spectrum disorder (ASD) (OR = 0.32; *p* = 0.041). Protective effects were found for 4–8 months of breastfeeding limited to specific subscales. No additional benefits were observed beyond 8 months.

*Conclusions*: Early breastfeeding, particularly within the first 4 months, may protect effects against emotional and behavioral problems, suggesting that intensity rather than prolonged durations could be more relevant for child development.

**Table:** 

**What is Known:** • *Any breastfeeding has been associated with positive effects on child development, including possible protection against behavioral and emotional problems.* • *Evidence on the duration of any breastfeeding and its impact on mental health outcomes in early childhood remains inconsistent.* **What is New:** • *Breastfeeding during the first 4 months is associated with fewer internalizing and externalizing problems at 4 years.* • *Protective effects diminish beyond 8 months, suggesting intensity in the early period may be more relevant than prolonged duration.*

**What is Known:**

• *Any breastfeeding has been associated with positive effects on child development, including possible protection against behavioral and emotional problems.*

• *Evidence on the duration of any breastfeeding and its impact on mental health outcomes in early childhood remains inconsistent.*

**What is New:**

• *Breastfeeding during the first 4 months is associated with fewer internalizing and externalizing problems at 4 years.*

• *Protective effects diminish beyond 8 months, suggesting intensity in the early period may be more relevant than prolonged duration.*

## Background

Breastfeeding benefits for child health are well-documented [1], including strengthened immunity, healthier gut microbiota, optimal neurodevelopment, and improved cognitive outcomes [2–7]. In contrast, its potential role in psychological and behavioral development has been less extensively investigated, and findings to date remain inconsistent [8, 9].

Some investigations have observed positive associations between breastfeeding and lower incidence of childhood behavioral problems [10–12], whereas other studies have found no conclusive results [13], or reported that the associations disappear after adjusting for confounding variables [14]. Together, these findings suggest a complex relationship, indicating that breastfeeding’s potential benefits on behavioral outcomes may depend on biological, social, and environmental moderators.

The systematic review conducted by Poton et al. [12] suggested that children who were breastfed for at least 4 months exhibited fewer behavioral problems. However, the relationship with emotional problems remains unclear due to the limited number of studies. Since then, newer longitudinal studies have provided mixed results, reinforcing both the possibility of protective effects and the importance of contextual factors. For instance, Boucher et al. [11] found that a longer duration of breastfeeding was associated with a slight reduction in autistic traits and, to a lesser extent, attention deficit and hyperactivity disorder (ADHD) symptoms. However, these effects were relatively weak and of limited clinical relevance. Lamma et al. [8] found no significant association between breastfeeding duration and emotional, behavioral, or hyperactivity problems at age 5, while Girard et al. [15] reported lower emotional reactivity and somatic complaints with breastfeeding for at least 6 months, but no further benefits beyond 12 months.

Similarly, Soled et al. [16], after adjusting for multiple confounding variables, found that exclusive breastfeeding for at least 6 months was associated with a lower likelihood of preschool ADHD, specifically an 8% reduction in risk for each additional month of breastfeeding. In fact, two recent meta-analyses concluded that not being breastfed was a significant risk factor for receiving a diagnosis of ADHD [17] and autism [18], findings consistent with those reported by Huang et al. [19].

More recently, Meng et al. [20] found that both exclusive breastfeeding during the first 6 months and prolonged breastfeeding (up to 18 months) were associated with fewer internalizing and externalizing problems, while Turner et al. [21] emphasized that the benefits of breastfeeding beyond 18 months appeared to be mediated by the quality of the mother–child relationship and maternal emotional state. Complementarily, the UK Millennium Cohort Study [9] also indicated a consistent relationship between breastfeeding (up to 9 months) and lower behavioral problems scores at various ages, even after adjusting for numerous sociodemographic factors, although it highlighted that the benefits were not uniform across developmental stages.

Taken together, these findings suggest that breastfeeding may have a protective effect on children’s emotional and behavioral development. Nevertheless, the evidence remains inconclusive, partly due to sample heterogeneity, methodological differences, and variability in the ages at which psychological problems are assessed. Moreover, the lack of consensus on how to define and measure breastfeeding (exclusive, mixed, or partial), as well as the diversity in cutoff points used to determine its duration and the limited consideration of contextual factors, complicates comparison across studies. Given these inconsistencies, research that jointly examines breastfeeding duration and contextual variables is needed to isolate the association between breastfeeding and children’s mental health.

In light of this, the present study aims to investigate the association between breastfeeding duration and emotional and behavioral problems in children at age 4. Based on the reviewed evidence, we hypothesize that children who were breastfed during the first months of life will show a lower probability of exhibiting emotional and behavioral symptoms during early childhood. Furthermore, we expect to observe a differential effect depending on the duration of breastfeeding.

## Methods

### Selection and description of participants

**Fig. 1:**
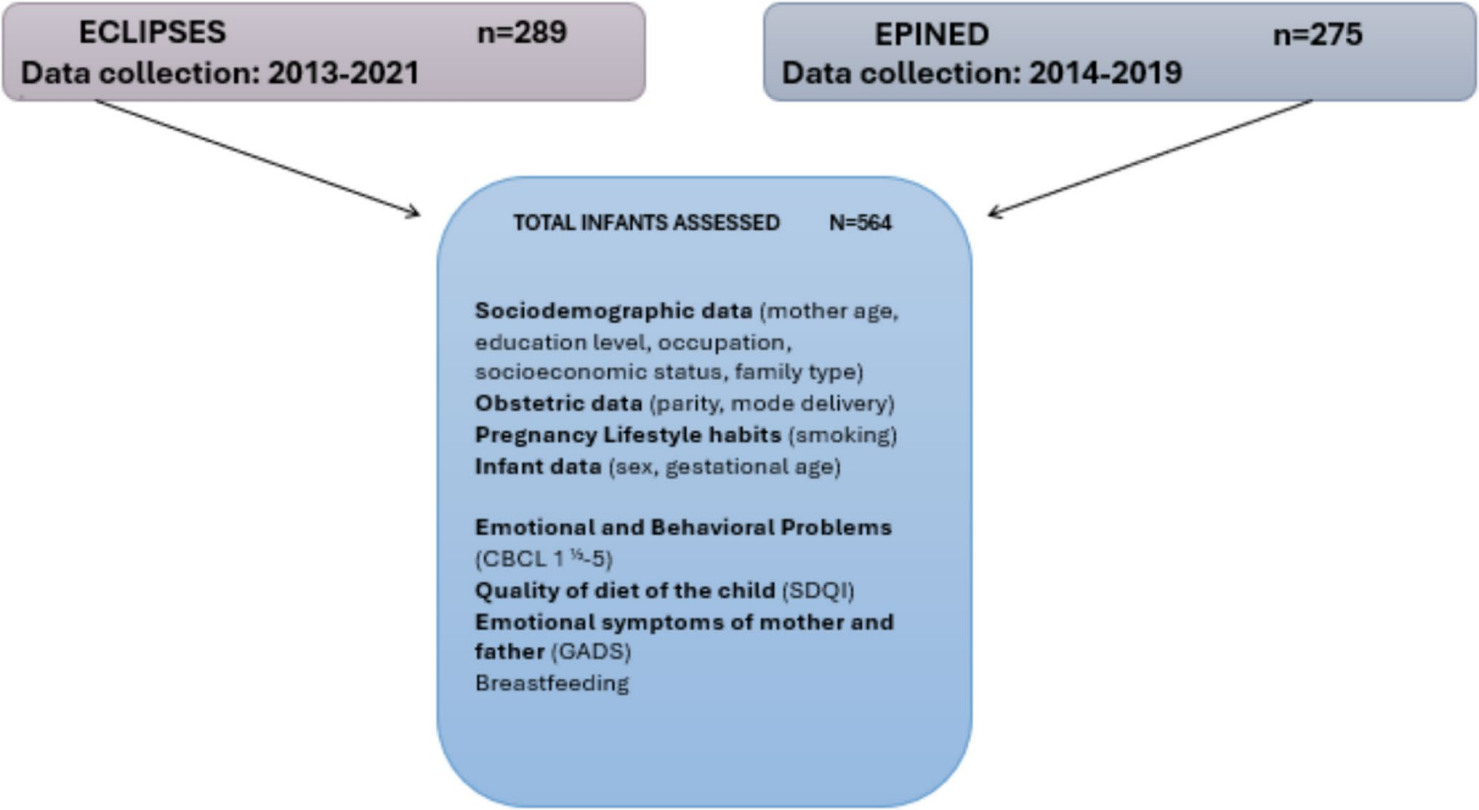
Selection and description of participants

The sample (*n* = 564) consisted of participants from both the ECLIPSES study (*n* = 289) [22] and the EPINED study [23] (*n* = 275), conducted in the Tarragona region (Catalonia, Spain) (Fig. 1).

The ECLIPSES study was a community-based research study conducted with pregnant women and their infants with the aim to explore the impact of nutritional, environmental, and sociodemographic factors during pregnancy and child development. The study design of ECLIPSES included three antenatal visits (at 12, 24, and 36 weeks), a postpartum visit at 40 days after delivery, and a follow-up 4 years later. During these visits, sociodemographic, clinical, and psychosocial information was collected. Of the 793 women recruited at 12 weeks of gestation, 289 mothers and their children completed the 4-year follow-up. Participant attrition was due to voluntary withdrawal, newly emerging exclusion criteria during pregnancy, miscarriage, and loss to follow-up.

The EPINED study was a two-phase, cross-sectional study aimed at estimating the prevalence of autism spectrum disorder (ASD) and ADHD in a community-based school population sample, focusing on the influence of prenatal, perinatal, and postnatal factors. In the first phase, 3727 parents and teachers participated in a screening process, In the second phase, children with high screening scores for ASD and ADHD, along with a comparison group without risk, underwent detailed clinical and cognitive assessments. This phase included 781 children, of whom 275 were aged 4 and included in the present study.

### Data collection and measurement

#### Main measures

Breastfeeding information was collected from parents when children were 4 years old. Parents were asked to report the total number of months their child was breastfed, considering both exclusive breastfeeding and breastfeeding combined with formula feeding. For analysis, children were classified into four groups according to any breastfeeding duration: no breastfeeding, breastfeeding for 1–4 months, 4–8 months, and more than 8 months.

Emotional and behavioral problems were assessed using the Child Behavior Checklist for Ages 1^½^–5 (CBCL 1^½^–5) [24]. The CBCL 1^½^–5 consists of 99 items describing child behaviors over the past 2 months, rated by primary caregivers on a 3-point Likert scale. Scores are grouped into syndrome scales (emotionally reactive, anxious/depressed, somatic complaints, withdrawn, sleep problems, attention problems, aggressive behavior); broad problem scales (internalizing, externalizing, total problems); and DSM-oriented scales (depressive, anxiety, autism spectrum, attention/hyperactivity, oppositional defiant problems). Raw scores are converted to *T*-scores (mean = 50, SD = 10). Two outcome types were used: continuous scores, providing a dimensional assessment of symptoms; and dichotomous scores, based on clinical cutoffs (*T* ≥ 70 for syndrome and DSM scales; *T* ≥ 65 for broad problem scales), indicating clinically significant problems.

#### Adjustment measures

Obstetric and neonatal data were obtained from medical records at birth (ECLIPSES) and parental interviews when the child was 4 years old (EPINED), including maternal age, infant sex, gestational age, and birth weight.

Socioeconomic status (SES) was determined using the Hollingshead Index [25], combining parental education and occupation classified according to the Catalan occupational system. This information was obtained during pregnancy and confirmed in the 4-year-old’s follow-up in the ECLIPSES cohort and in the parents’ interview when the child was 4 years old in the ECLIPSES cohort.

Smoking during pregnancy was assessed during pregnancy with the Fagerström Questionnaire (Fagerström_Q) [26] in the ECLIPSES cohort and with ad hoc parental report when the child was 4 years old in the EPINED cohort. The information was coded as smoker and non-smoker.

Family structure information was obtained when the child was 4 years old in both cohorts. The information was coded as nuclear (parents and child living together) or other.

Parental emotional symptoms were assessed when the child was 4 years old in both cohorts using the Goldberg Anxiety and Depression Scale (GADS) [27], which yields separate scores for the presence or absence of clinically relevant anxiety and depression symptoms. For the present analysis, a dichotomous variable was created indicating the presence of emotional symptoms when parents screened positive for anxiety and/or depression.

Child diet quality at age 4 was assessed with the Standardized Diet Quality Index [28] in both cohorts, which considers nutrition variety, frequency, quantity, and adequacy to provide an overall score.

### Statistics

Prior to categorical analyses, breastfeeding duration (months) was examined both as a continuous variable and through exploratory percentile-based groupings to evaluate potential linear and non-linear patterns. These preliminary analyses indicated that associations were concentrated within the earliest months of breastfeeding. Accordingly, breastfeeding duration was categorized into four groups for the main analyses: no breastfeeding, 1–4 months, 4–8 months, and more than 8 months.

Descriptive analyses were then conducted for maternal and child characteristics. Differences by cohort (ECLIPSES, EPINED) and breastfeeding category (none, 1–4, 4–8, > 8 months) were tested using Student’s *t*-test/ANOVA for continuous variables and *χ*^2^ tests for categorical variables. Associations between breastfeeding and CBCL 1^½^–5 scores were examined using unadjusted and adjusted multiple linear regression models (enter method). Adjusted models included mother’s age at birth (years), family socioeconomic status (low/medium/high), maternal smoking during pregnancy (yes/no), infant sex (girl/boy), gestational age at birth (weeks), family type (nuclear/other), maternal emotional symptoms (presence/absence), paternal emotional symptoms (presence/absence), quality of child’s diet (score), and cohort (EPINED/ECLIPSES). Logistic regression models (unadjusted and adjusted) were used to estimate odds of clinical-range CBCL 1^½^–5 scores with the same covariates. Collinearity and interaction terms were tested but were nonsignificant.

## Results

### Descriptive data of the sample

Descriptive characteristics and cohort differences are shown in Table 1. The mean maternal age was 33.61 years (SD = 5.6); most families had medium socioeconomic status, and 87.0% were nuclear at assessment. Overall, 44.5% of infants were girls, with a significantly higher proportion of boys in EPINED (60.7%) than girls (39.3%) (*χ*^2^ = 6.256, *p* = 0.012). Among prenatal factors, 82.9% of mothers reported not smoking during pregnancy, and mean gestational age was 39.31 weeks (SD = 2.1). Any breastfeeding prevalence was 72.7%, with a mean duration of 9.27 months (SD = 11.8), significantly shorter in EPINED (6.87 months, SD = 10.3) than in ECLIPSES (11.54 months, SD = 12.7) (*t* = 4.804; *p* < 0.001). Among breastfeeding mothers, 20.7% breastfed for 1–4 months, 16.9% for 4–8 months, and 35.1% for > 8 months, with ECLIPSES showing the highest proportion in this category (*χ*^2^ = 24.639, *p* < 0.001).

**Table 1:** Descriptive characteristics of the sample and differences by cohort

	Total sample *n* = 564	EPINED cohort *n* = 275	ECLIPSES cohort *n* = 289	
Data collection		2014–2019	2013–2021	
	Mean (SD)*	Mean (SD)*	Mean (SD)*	*t* (*p*)*
	*n* (%)^#^	*n* (%)^#^	*n* (%)^#^	*X*^2^ (*p*)^#^
Mothers’ age (years)*	33.61 (5.7)	35.90 (5.7)	31.54 (4.7)	102.937 (< 0.001)
Family socioeconomic status^#^				
Low	174 (28.4)	90 (30.5)	84 (26.4)	1.584 (0.453)
Medium	346 (56.4)	164 (55.6)	182 (57.2)	
High	93 (15.2)	41 (13.9)	52 (16.4)	
Pregnancy mother tobacco use (no)^#^	470 (82.9)	211 (83.1)	259 (82.7)	0.010 (0.919)
Infant sex (girls)^#^	273 (44.5)	157 (49.4)	116(39.3)	6.256 (0.012)
Gestational age (weeks)*	39.31 (2.1)	38.82 (2.6)	39.75 (1.4)	25.627 (< 0.001)
Family type (nuclear)^#^	504 (87.0)	238 (80.7)	266 (93.7)	21.634 (< 0.001)
Mother emotional symptoms (absence)^#^	283 (54.2)	198 (70.0)	85 (35.6)	61.171 (< 0.001)
Father emotional symptoms (absence)^#^	296 (62.8)	186 (75.6)	110 (48.9)	35.934 (< 0.001)
Quality of children's diet (score)*	61.70 (10.0)	62.54 (7.4)	60.93 (10.8)	4.570 (0.033)
Any breastfeeding (months)*	9.27 (11.8)	6.87 (10.3)	11.54 (12.7)	22.840 (< 0.001)
Not breastfeeding^#^	154 (27.3)	94 (34.2)	60 (20.8)	24.639 (< 0.001)
Any breastfeeding from 1 to 4 months^#^	117 (20.7)	67 (24.4)	50 (17,3)	
Any breastfeeding from 4 to 8 months^#^	95 (16.9)	40 (14.5)	55 (19,0)	
Any breastfeeding more than 8 months^#^	198 (35.1)	74 (26,9)	124 (42,9)	

^*^Results showed as mean and standard deviation (SD)

^#^Results showed as *n* and percentage (%)

Descriptive data of CBCL 1^½^–5 scores and cohort differences are shown in Table 2. EPINED infants scored significantly higher on all syndromic scales than those from ECLIPSES and showed a higher proportion of children at risk on DSM-oriented scales.

**Table 2:** Descriptive data of CBCL 1^½^–5 scores for the total sample and differences by cohort

		Total sample *n* = 564	EPINED Cohort *n* = 275	ECLIPSES Cohort *n* = 289	
		Mean (SD)*	Mean (SD)*	Mean (SD)*	*t* (*p*)*
		*n* (%)^#^	*n* (%)^#^	*n* (%)^#^	*X*^2^ (*p*)#
Syndrome scales					
Emotionally reactive	Total score*	58.29 (9.9)	59.27 (9.9)	57.07 (9.7)	6.444 (0.011)
	≥ 70 score^#^	82 (15.9)	54 (18.9)	28 (12.1)	4.375 (0.036)
Anxiety depression	Total score*	57.44 (8.3)	58.61 (8.2)	55.98 (8.2)	13.283 (< 0.001)
	≥ 70 score^#^	43 (8.3)	29 (10.1)	14 (6.1)	2.789 (0.095)
Somatic complaints	Total score*	56.23 (7.3)	57.00 (7.1)	55.26 (7.6)	7.269 (0.007)
	≥ 70 score^#^	34 (6.6)	22 (7.7)	12 (5.2)	1.297 (0.255)
Withdrawn	Total score*	59.45 (9.3)	60.66 (9.7)	57.96 (8.6)	10.996 (< 0.001)
	≥ 70 score^#^	84 (16.2)	58 (20.3)	26 (11.3)	7.648 (0.006)
Sleep problems	Total score*	55.66 (7.1)	55.80 (6.8)	55.48 (7.6)	0.256 (0.613)
	≥ 70 score^#^	30 (5.8)	19 (6.6)	11 (4.8)	0.828 (0.363)
Attention problems	Total score*	59.19 (7.9)	60.45 (7.5)	57.63 (8.1)	16.710 (< 0.001)
	≥ 70 score^#^	52 (10.1)	35 (12.2)	17 (7.4)	3.362 (0.067)
Aggressive behavior	Total score*	57.39 (8.7)	59.01 (9.1)	55.39 (7.8)	22.761 (< 0.001)
	≥ 70 score^#^	53 (10.3)	38 (13.3)	15 (6.5)	6.410 (0.011)
Broad-band scales					
Internalizing problems	Total score*	57.38 (12.2)	59.40 (11.9)	54.88 (12.2)	17.981 (< 0.001)
	≥ 65 score^#^	134 (25.9)	92 (32.2)	42 (18.2)	13.018 (< 0.001)
Externalizing problems	Total score*	56.25 (12.0)	58.61 (12.0)	53.32 (11.3)	26.028 (0.001)
	≥ 65 score^#^	112 (21.7)	83 (29.0)	29 (12.6)	20.418 (< 0.001)
Total problems	Total score*	57.80 (12.7)	60.27 (12.6)	54.74 (12.2)	25.134 (0.001)
	≥ 65 score^#^	148 (28.6)	102 (35.7)	46 (19.9)	15.516 (< 0.001)
DSM oriented scales					
Depressive problems	Total score*	58.61 (8.5)	60.18 (8.4)	56.66 (8.3)	22.660 (< 0.001)
	≥ 70 score^#^	55 (10.6)	42 (14.7)	13 (5.6)	11.028 (< 0.001)
Anxiety problems	Total score*	58.42 (8.6)	59.31 (8.3)	57.32 (8.9)	6.951 (0.009)
	≥ 70 score^#^	62 (12.0)	41 (14.3)	21 (9.1)	3.331 (0.068)
Autism problems	Total score*	59.02 (8.9)	60.31 (9.1)	57.42 (8.4)	13.791 (< 0.001)
	≥ 70 score^#^	71 (13.7)	51 (17.8)	20 (8.7)	9.078 (0.003)
Attention deficit	Total score*	59.16 (8.7)	60.81 (8.6)	57.11 (8.5)	23.908 (< 0.001)
Hyperactivity problems	≥ 70 score^#^	75 (14.5)	51 (17.8)	24 (10.4)	5.707 (0.017)
Oppositional defiant problems	Total score*	55.88 (7.8)	56.87 (7.9)	54.64 (7.4)	10.745 (0.001)
	≥ 70 score^#^	48 (9.3)	33 (11.5)	15 (6.5)	3.862 (0.049)

^*^Results showed as mean and standard deviation (SD)

^#^Results showed as *n* and percentage (%)

Table 3 presents sociodemographic and perinatal variables by any breastfeeding group. Mothers who breastfed were less likely to smoke during pregnancy (*χ*^2^ = 21.178, *p* < 0.001), had longer gestations (*F* = 3.017, *p* = 0.030), and had higher SES (*F* = 17.528, *p* = 0.008) compared with non-breastfeeding mothers. No significant differences were found in other sociodemographic variables.

**Table 3:** Descriptive characteristics of the sample according to any breastfeeding groups

	No breastfeeding *n* = 154	Any breastfeeding from 1 to 4 months *n* = 117	Any breastfeeding from 4 to 8 months *n* = 95	Any breastfeeding more than 8 months *n* = 198	
	Mean (SD)*	Mean (SD)*	Mean (SD)*	Mean (SD)*	*F*(*p*)* X^2^ (p)^#^
	*n* (%)^#^	*n* (%)^#^	*n* (%)^#^	*n* (%)^#^	
Mothers’ age (years)*	29.73 (6.1)	29.03 (5.5)	28.38 (4.8)	28.64 (5.2)	1.590 (0.191)
Family socioeconomic status^#^					
Low	43 (26.5)	35 (21.6)	17 (10.5)	67 (41.4)	17.528 (0.008)
Medium	94 (30.3)	69 (22.0)	56 (17.9)	94 (30.0)	
High	17 (19.1)	13 (14.6)	22 (24.7)	37 (41.6)	
Pregnancy mother tobacco use (no)^#^	99 (22.7)	90 (20.6)	81 (18.5)	167 (38.2)	21.178 (< 0.001)
Infant sex (girls)^#^	69 (27.1)	56 (22.0)	36 (14.1)	94 (36.9)	2.805 (0.423)
Gestational age (weeks)*	38.91 (2.4)	39.12 (2.5)	39.5 (1.6)	39.59 (1.8)	3.017 (0.030)
Family type (nuclear)^#^	123 (25.3)	103 (21.1)	85 (17.5)	176 (36.1)	4.926 (0.177)
Mother emotional symptoms (absence)^#^	70 (26.3)	60 (22.6)	47 (17.7)	89 (33.5)	0.969 (0.809)
Father emotional symptoms (absence)^#^	80 (28.6)	63 (22.5)	45 (16.1)	92 (32.9)	1.588 (0.662)
Quality of children’s diet (score)*	61.15 (8.6)	61.07 (8.2)	62.91 (8.5)	63.01 (10.0)	1.996 (0.113)

^*^ Results showed as mean and standard deviation (SD)

^#^ Results showed as *n* and percentage (%)

Table 4 shows CBCL 1^½^–5 continuous scores and proportion of clinical-risk scores by any breastfeeding group. No significant differences were found in continuous scores. However, infants breastfed > 8 months had higher rates on somatic complaints (*χ*^2^ = 10.866, *p* = 0.012), withdrawn (*χ*^2^ = 10.298, *p* = 0.016), and aggressive behavior (*χ*^2^ = 9.578, *p* = 0.023).

**Table 4:** CBCL 1^½^–5 scores according to any breastfeeding groups

		No breastfeeding *n* = 154	Any breastfeeding from 1 to 4 months *n* = 117	Any breastfeeding from 4 to 8 months *n* = 95	Any breastfeeding more than 8 months *n* = 198	
		Mean (SD)*	Mean (SD)*	Mean (SD)*	Mean (SD)*	*F* (*p*)*
		*n* (%)^#^	*n* (%)^#^	*n* (%)^#^	*n* (%)^#^	*X*^2^ (*p*)^#^
Syndrome scales						
Emotionally reactive	Total score*	59.02 (9.7)	57.17 (10.4)	57.99 (10.3)	58.34 (9.5)	0.724 (0.538)
	≥ 70 score^#^	21 (26.9)	17 (21.8)	15 (19.2)	25 (32.1)	0.429 (0.934)
Anxiety depression	Total score*	58.37 (7.8)	56.57 (9.1)	56.29 (6.8)	57.81 (8.7)	1.623 (0.183)
	≥ 70 score^#^	14 (33.3)	6 (14.3)	4 (9.5)	18 (42.9)	4.188 (0.242)
Somatic complaints	Total score*	56.88 (7.3)	54.92 (7.7)	55.31 (6.3)	56.72 (7.3)	2.207 (0.086)
	≥ 70 score^#^	12 (37.5)	4 (12.5)	0 (0.0)	16 (50.0)	10.866 (0.012)
Withdrawn	Total score*	60.19 (9.6)	57.68 (8.8)	58.18 (7.6)	59.94 (9.7)	2.215 (0.086)
	≥ 70 score^#^	28 (36.4)	10 (13.0)	7 (9.1)	32 (41.6)	10.298 (0.016)
Sleep problems	Total score*	55.49 (6.3)	55.49 (6.3)	55.72 (7.7)	56.14 (6.7)	0.449 (0.718)
	≥ 70 score^#^	7 (25.0)	5 (17.9)	5 (4.7)	11 (39.3)	0.575 (0.902)
Attention problems	Total score*	60.05 (7.6)	57.78 (9.2)	58.67 (7.5)	59.4 (7.5)	1.8445 (0.138)
	≥ 70 score^#^	14 (28.0)	7 (14.0)	10 (20.0)	19 (38.0)	2.097 (0.553)
Aggressive behavior	Total score*	58.25 (8.7)	56.14 (8.6)	56.42 (7.6)	57.81 (9.6)	1.611 (0.186)
	≥ 70 score^#^	20 (39.2)	3 (5.9)	8 (15.7)	20 (39.2)	9.578 (0.023)
Broad-band scales						
Internalizing problems	Total score*	58.67 (12.1)	55.78(11.8)	55.83 (11.6)	57.75 (12.7)	1.603 (0.188)
	≥ 65 score^#^	39 (31.5)	26 (21.0)	15 (12.1)	44 (35.5)	3.102 (0.376)
Externalizing problems	Total score*	57.43 (11.9)	55.09 (11.9)	54.71 (11.7)	56.56 (12.4)	1.248 (0.292)
	≥ 65 score^#^	35(32.7)	23 (21.5)	15 (14.0)	34 (31.8)	1.877 (0.598)
Total problems	Total score*	59.06 (12.8)	56.55 (11.6)	56.00 (11.7)	58.04 (13.4)	1.341 (0.260)
	≥ 65 score^#^	43 (30.9)	29 (20.9)	19 (13.7)	48 (34.5)	1.831 (0.608)
DSM oriented scales						
Depressive problems	Total score*	59.02 (7.9)	58.21 (9.6)	56.84 (8.0)	58.9 (8.6)	1.424 (0.235)
	≥ 70 score^#^	14 (27.5)	8 (15.7)	8 (15.7)	21 (41.2)	1.897(0.594)
Anxiety problems	Total score*	59.01 (8.0)	57.93 (10.0)	57.58 (7.6)	58.74 (8.7)	0.665 (0.574)
	≥ 70 score^#^	17 (28.8)	13 (22.0)	9 (15.3)	20 (33.9)	0.126 (0.989)
Autism problems	Total score*	59.90 (8.9)	57.28 (8.7)	58.28 (7.8)	59.20 (9.2)	1.983 (0.116)
	≥ 70 score^#^	20 (32.3)	8 (12.9)	8 (12.9)	26 (41.9)	4.989 (0.173)
Attention deficit	Total score*	60.28 (8.8)	57.79 (9.6)	58.20 (8.0)	59.34 (8.5)	1.951 (0.120)
Hyperactivity problems	≥ 70 score^#^	24 (32.9)	13 (17.8)	9 (12.3)	27 (37.0)	2.587 (0.460)
Oppositional defiant problems	Total score*	56.59 (7.8)	55.39 (8.7)	54.73 (6.5)	56.20 (7.7)	1.216 (0.303)
	≥ 70 score^#^	16 (34.0)	6 (12.8)	7 (14.9)	18 (38.3)	2.970 (0.396)

^*^Results showed as mean and standard deviation (*SD*)

^#^Results showed as *n* and percentage (%)

### Predictive relationship between any breastfeeding and child behavior

Table 5 shows unadjusted and adjusted multiple linear regression models linking breastfeeding groups to CBCL 1^½^–5 scores. Significant associations were observed between non-breastfed children and those breastfed for 1–4 months.

**Table 5:** Unadjusted and adjusted linear regression models for CBCL 1^1/2^–5 scales according to any breastfeeding groups (*n* = 564)

	No breastfeeding vs any breastfeeding from 1 to 4 months	*p*	No breastfeeding vs any breastfeeding from 4 to 8 months	*p*	No breastfeeding vs any breastfeeding more than 8 months	*p*
	*B* (95% confidence interval)		*B* (95% confidence interval)		*B* (95% confidence interval)	
Syndrome scales						
Emotionally reactive						
Unadjusted model	− 1.85 (− 4.36 to 0.66)	0.148	− 1.03 (− 3.73 to 1.67)	0.452	− 0.68 (− 2.92 to 1.55)	0.548
Adjusted model	− 3.07 (− 5.83 to − 0.31)	0.029	− 1.06 (− 4.106 to 1.994)	0.496	− 1.20 (− 3.72 to 1.32)	0.349
Anxiety depression						
Unadjusted model	− 1.80 (− 3.90 to 0.29)	0.092	− 2.08 (− 4.34 to 0.18)	0.071	− 0.56 (− 2.43 to 1.32)	0.560
Adjusted model	− 2.51 (− 4.75 to − 0.27)	0.028	− 1.67 (− 4.14 to 0.81)	0.186	− 0.95 (− 2.99 to 1.10)	0.363
Somatic complaints						
Unadjusted model	− 1.96 (− 3.80 to − 0.12)	0.036	− 1.56 (− 3.54 to 0.41)	0.121	− 0.15 (− 1.79 to 1.49)	0.854
Adjusted model	− 2.51 (− 4.48 to − 0.53)	0.013	− 0.73 (− 2.91 to 1.45)	0.511	− 0.39 (− 2.19 to 1.42)	0.674
Withdrawn						
Unadjusted model	− 2.51 (− 4.83 to − 0.19)	0.034	− 2.01 (− 4.51 to 0.49)	0.115	− 0.25 (− 2.32 to 1.82)	0.814
Adjusted model	− 2.85 (− 5.45 to − 0.26)	0.031	− 2.19 (− 5.06 to 0.67)	0.133	− 0.64 (− 2.99 to 1.74)	0.604
Sleep problems						
Unadjusted model	− 0.33 (− 2.15 to 1.49)	0.720	− 0.23 (− 1.73 to 2.19)	0.818	0.65 (− 0.97 to 2.28)	0.431
Adjusted model	− 0.58 (− 2.50 to 1.33)	0.550	0.31 (− 1.80 to 2.43)	0.770	− 0.08 (− 1.82 to 1.67)	0.932
Attention problems						
Unadjusted model	− 2.27 (− 4.28 to − 0.26)	0.027	− 1.38 (− 3.54 to 0.79)	0.035	− 0.57 (− 2.36 to 1.22)	0.533
Adjusted model	− 2.04 (− 4.10 to 0.01)	0.051	− 0.63 (− 2.90 to 1.64)	0.585	− 0.11 (− 1.99 to 1.76)	0.905
Aggressive behavior						
Unadjusted model	− 2.11 (− 4.34 to 0.12)	0.063	− 1.83 (− 4.23 to 0.57)	0.134	− 0.45 (− 2.43 to 1.54)	0.660
Adjusted model	− 2.72 (− 5.00 to − 0.43)	0.020	− 1.89 (− 4.42 to 0.63)	0.141	− 1.13 (− 3.22 to 0.95)	0.286
Broad-band scales						
Internalizing problems						
Unadjusted model	− 2.88 (− 5.98 to 0.21)	0.068	− 2.83 (− 6.16 to 0.49)	0.095	− 0.91 (− 3.67 to 0.85)	0.515
Adjusted model	− 4.21 (− 7.58 to − 0.84)	0.014	− 2.24 (− 5.96 to 1.47)	0.236	− 1.32 (− 4.41 to 1.73)	0.391
Externalizing problems						
Unadjusted model	− 2.33 (− 5.39 to 0.72)	0.134	− 2.72 (− 6.00 to 0.57)	0.105	− 0.87 (− 3.59 to 1.86)	0.532
Adjusted model	− 3.30 (− 6.51 to − 0.09)	0.044	− 2.42 (− 5.97 to 1.13)	0.180	− 1.12 (− 4.05 to 1.81)	0.454
Total problems						
Unadjusted model	− 2.51 (− 5.75 to 0.73)	0.128	− 3.06 (− 6.54 to 0.42)	0.085	− 1.02 (− 3.90 to 1.87)	0.490
Adjusted model	− 4.13 (− 7.56 to − 0.69)	0.019	− 2.42 (− 6.22 to 1.38)	0.211	− 1.38 (− 4.52 to 1.76)	0.388
DSM Oriented scales						
Depressive problems						
Unadjusted model	− 0.81 (− 2.97 to 1.35)	0.459	− 2.18 (− 4.50 to 0.14)	0.066	− 0.06 (− 1.98 to 1.87)	0.953
Adjusted model	− 1.85 (− 4.11 to 0.41)	0.108	− 1.78 (− 4.27 to 0.72)	0.162	− 0.5 (− 2.56 to 1.56)	0.635
Anxiety problems						
Unadjusted model	− 1.08 (− 3.28 to 1.12)	0.334	− 1.44 (− 3.80 to 0.93)	0.233	− 0.27 (− 2.23 to 1.69)	0.784
Adjusted model	− 2.23 (− 4.61 to 0.15)	0.066	− 1.66 (− 4.29 to 0.97)	0.214	− 1.04 (− 3.21 to 1.14)	0.349
Autism problems						
Unadjusted model	− 2.62 (− 4.85 to − 0.38)	0.022	− 1.62 (− 4.02 to 0.78)	0.185	− 0.69 (− 2.68 to 1.30)	0.494
Adjusted model	− 2.74 (− 5.18 to − 0.30)	0.028	− 1.85 (− 4.55 to 0.85)	0.178	− 0.97 (− 3.20 to 1.26)	0.393
Attention deficit hyperactivity problems						
Unadjusted model	− 2.48 (− 4.70 to − 0.26)	0.028	− 2.07 (− 4.46 to 0.32)	0.089	− 0.93 (− 2.91 to 1.05)	0.356
Adjusted model	− 2.82 (− 5.17 to − 0.46)	0.019	− 1.78 (− 4.38 to 0.82)	0.180	− 1.18 (− 3.33 to 0.97)	0.280
Oppositional defiant problems						
Unadjusted model	− 1.21 (− 3.19 to 0.77)	0.231	− 1.86 (− 3.99 to 0.27)	0.087	− 0.395 (− 2.16 to 1.37)	0.660
Adjusted model	1.50 (− 3.62 to 0.62)	0.164	− 1.62 (− 3.96 to 0.72)	0.175	− 0.62 (− 2.55 to 1.31)	0.529

Models adjusted for the following: mothers’ age (years); family socioeconomic status (low, medium, high); pregnancy mother tobacco use (no, yes); infant sex (girl, boy); gestational age (weeks); family type (nuclear, other configuration); mother emotional symptoms (absence, presence); father emotional symptoms (absence, presence); quality of children’s diet (score), cohort (EPINED, ECLIPSES)

In unadjusted models, any breastfeeding up to 4 months was linked to lower scores on somatic complaints (*β* = − 1.96, 95%CI = − 3.80 to − 0.12, *p* = 0.036), withdrawn (*β* = –2.51, 95%CI = –4.83 to − 0.19, *p* = 0.034), and attention problems (*β* = –2.27, 95%CI = –4.28 to − 0.26, *p* = 0.03), as well as lower DSM-oriented autism (*β* = –2.62, 95%CI = –4.85 to − 0.38, *p* = 0.022) and ADHD problems (*β* = –2.48, 95%CI = –4.70 to − 0.26, *p* = 0.028) scales.

After adjustment, any breastfeeding for ≤ 4 months remained associated with lower scores on emotionally reactive (*β* = –3.07, 95%CI = –5.83 to − 0.31, *p* = 0.029), attention problems (*β* = –2.04, 95%CI = –4.10 to − 0.01, *p* = 0.051), somatic complaints (*β* = –2.51, 95%CI = –4.48 to − 0.53, *p* = 0.013), withdrawn (β = –2.85, 95%CI = –5.45 to − 0.26, *p* = 0.031), and aggressive behavior (β = –2.72, 95%CI = –5.00 to − 0.43, *p* = 0.020).

Broad-band scales also showed lower internalizing (*β* = –4.21, 95%CI = –7.58 to − 0.84, *p* = 0.014), externalizing (*β* = –3.30, 95%CI = –6.51 to − 0.09, *p* = 0.044), and total problems (*β* = –4.13, 95%CI = –7.56 to − 0.69, *p* = 0.019). Similar reductions were observed for DSM-oriented autism (*β* = –2.74, 95%CI = –5.18 to − 0.30, *p* = 0.028) and ADHD problems (*β* = –2.82, 95%CI = –5.17 to − 0.46, *p* = 0.019) scales.

Table 6 shows logistic regression models linking any breastfeeding duration to clinical risk scores on CBCL 1^½^–5 scales. Compared with non-breastfed children, any breastfeeding for 1–4 months was associated with lower risk on withdrawn (OR = 0.21, *p* = 0.004), somatic complaints (OR = 0.19, *p* = 0.048), aggressive behavior (OR = 0.08, *p* = 0.016), and internalizing problems (OR = 0.35,* p* = 0.010). A similar association was observed for 4–8 months of any breastfeeding on withdrawn (OR = 0.25, *p* = 0.022).

**Table 6:** Unadjusted and adjusted logistic regression models for clinical CBCL 1^1/2^–5 scale scores according to any breastfeeding groups (*n* = 564)

	No breastfeeding vs any breastfeeding from 1 to 4 months *n* = 271	*p*	No breastfeeding vs any breastfeeding from 4 to 8 months *n* = 249	*p*	No breastfeeding vs any breastfeeding more than 8 months *n* = 352	*p*
	Odds ratio (95% confidence interval)		Odds ratio (95% confidence interval)		Odds ratio (95% confidence interval)	
Syndrome scales						
Emotionally reactive						
Unadjusted model	1.06 (0.53–2.14)	0.861	1.229 (0.594–2.542)	0.578	0.99 (0.53–1.85)	0.970
Adjusted model	0.49 (0.18–1.36)	0.171	1.24 (0.46–3.31)	0.669	0.91 (0.40–2.10)	0.833
Anxiety depression						
Unadjusted model	0.53 (0.14–1.41)	0.212	0.45 (0.14–1.41)	0.170	1.08 (0.52–2.25)	0.843
Adjusted model	0.34 (0.08–1.35)	0.124	0.43 (0.08–2.16)	0.302	1.15 (0.43–3.09)	0.776
Somatic complaints						
Unadjusted model	0.41 (0.130–1.32)	0.134	0.00 (0.00–0.00)	0.997	1.12 (0.51–2.46)	0.777
Adjusted model	0.19 (0.04–0.99)	0.048	0.00 (0.00–0.00)	0.997	0.72 (0.26–1.99)	0.525
Withdrawn						
Unadjusted model	0.41 (0.190–0.89)	0.023	0.36 (0.15–0.87)	0.023	0.94 (0.53–1.65)	0.825
Adjusted model	0.21 (0.08–0.61)	0.004	0.25 (0.08–0.82)	0.022	0.66 (0.31–1.38)	0.270
Sleep problems						
Unadjusted model	0.93 (0.29–3.01)	0.899	1.20 (0.37–3.91)	0.763	1.33 (0.50–3.52)	0.569
Adjusted model	0.95 (0.24–3.88)	0.947	1.19 (0.32–4.44)	0.793	0.82 (0.27–2.46)	0.718
Attention problems						
Unadjusted model	0.63 (0.24–1.61)	0.323	1.21 (0.51–2.87)	0.660	1.15 (0.55–2.38)	0.717
Adjusted model	0.27 (0.069–1.10)	0.067	1.48 (0.48–4.52)	0.490	0.85 (0.32–2.26)	0.743
Aggressive behavior						
Unadjusted model	0.17 (0.05–0.60)	0.005	0.63 (0.26–1.50)	0.297	0.81 (0.42–1.57)	0.531
Adjusted model	0.08 (0.01–0.62)	0.016	0.63 (0.20–1.97)	0.431	0.66 (0.26–1.69)	0.387
Broad-band scales						
Internalizing problems						
Unadjusted model	0.83 (0.46–1.47)	0.514	0.56 (0.29–1.10)	0.090	0.92 (0.55–1.52)	0.732
Adjusted model	0.35 (0.16–0.78)	0.010	0.50 (0.21–1.22)	0.128	0.78 (0.40–1.51)	0.462
Externalizing problems						
Unadjusted model	0.82 (0.45–1.49)	0.505	0.65 (0.33–1.28)	0.212	0.76 (0.44–1.30)	0.313
Adjusted model	0.61 (0.28–1.32)	0.208	0.74 (0.31–1.77)	0.495	0.54 (0.26–1.12)	0.098
Total problems						
Unadjusted model	0.83 (0.48–1.46)	0.519	0.66 (0.35–1.23)	0.187	0.90 (0.55–1.23)	0.671
Adjusted model	0.521(0.25–1.08)	0.081	0.55 (0.24–1.28)	0.163	0.79 (0.41–1.50)	0.467
DSM oriented scales						
Depressive problems						
Unadjusted model	0.72 (0.29–1.79)	0.484	0.95 (0.38–2.36)	0.903	1.28 (0.63–2.63)	0.496
Adjusted model	0.93 (0.31–2.83)	0.904	1.50 (0.43–5.30)	0.525	1.36 (0.49–3.78)	0.561
Anxiety problems						
Unadjusted model	0.99 (0.46–2.15)	0.990	0.87 (0.37–2.04)	0.742	0.98 (0.49–1.94)	0.943
Adjusted model	0.51 (0.18–1.44)	0.205	1.04 (0.34–3.14)	0.945	0.92 (0.38–2.23)	0.847
Autism problems						
Unadjusted model	0.48 (0.20–1.14)	0.097	0.63 (0.26–1.50)	0.297	1.10 (0.58–2.06)	0.777
Adjusted model	0.32 (0.11–0.96)	0.041	0.43 (0.13–1.44)	0.169	0.95 (0.42–2.14)	0.900
Attention deficit hyperactivity problems						
Unadjusted model	0.66 (0.32–1.38)	0.271	0.58 (0.25–1.31)	0.190	0.92 (0.51–169)	0.794
Adjusted model	0.54 (0.22–1.32)	0.175	0.60 (0.22–1.66)	0.327	0.61 (0.28–1.36)	0.225
Oppositional defiant problems						
Unadjusted model	0.46 (0.17–1.21)	0.116	0.70 (0.28–1.79)	0.458	0.93 (0.45–1.90)	0.836
Adjusted model	0.45 (0.13–1.57)	0.210	0.93 (0.28–3.19)	0.918	0.88 (0.32–2.41)	0.809

Models adjusted for the following: mothers’ age (years); family socioeconomic status (low, medium, high); pregnancy mother tobacco use (no, yes); infant sex (girl, boy); gestational age (weeks); family type (nuclear, other configuration); mother emotional symptoms (absence, presence); father emotional symptoms (absence, presence); quality of children’s diet (score), cohort (EPINED, ECLIPSES)

## Discussion

The results of this study indicate that any breastfeeding may serve as a protective factor for children’s mental health, particularly when breastfeeding is maintained during the first 4 months of life. At this time window, breastfed children showed reduced levels of emotional reactivity, anxiety/depression, somatic complaints, and withdrawn and aggressive behaviors, as well as lower scores on internalizing, externalizing, and total problems broad-band scales. Additionally, this group showed a reduced risk of reaching clinical-range scores on somatic complaints, withdrawn behavior, aggressive behavior, and internalizing problems, and exhibited a lower risk of ASD- and ADHD-related symptoms. Any breastfeeding up to eight months had a limited association with a decreased withdrawn behavior, and longer durations were not associated with additional benefits. However, slight increases in the proportion of clinical-range scores for somatic complaints and aggressive or withdrawn behavior were observed in children breastfed beyond 8 months. These effects were small and should be interpreted cautiously, but they underscore that the protective pattern appears strongest during the earliest months of breastfeeding.

These findings align with existing evidence reporting lower psychological problems in children breastfeed during early infancy [9, 10, 20]. However, although our original hypothesis suggested a linear cumulative protective association, our findings indicate that benefits are not progressively enhanced with longer breastfeeding durations, aligning with Lamma et al. [8], who question a sustained linear relationship between breastfeeding duration and childhood psychopathology. One possible explanation for the observed effects during the first 4 months is that this period coincides with maternity leave in our country, potentially reflecting a phase of more intensive breastfeeding and greater mother–infant contact. Moreover, complementary feeding is typically introduced around 6 months, which reduces the breastfeeding frequency and increases variability in the nutritional quality of complementary foods. In this context, the protective effect of breastfeeding on children’s mental health may be modulated by complementary diet quality and family feeding practices. In fact, a previous study has demonstrated that high consumption of free sugars during the early years is associated with increased behavioral and emotional problems [29]. Although this factor was not assessed in the present study or much of the existing literature, it may represent a noteworthy confounder potentially explaining the lack of additional benefits observed with extended breastfeeding. It is important to note, however, that our analyses did adjust for child dietary quality at age 4. Future studies should further explore whether changes in breastfeeding intensity, maternal return-to-work patterns, and complementary feeding practices during later infancy may help explain the weaker protective associations observed with longer breastfeeding durations.

On the other hand, much of the literature has focused on neurodevelopmental and behavioral disorders such as ADHD and ASD [11, 17–19], whereas fewer studies have addressed emotional or internalizing problems. In our study, children breastfed during the first 4 months exhibited significantly lower continuous and clinical scores in emotional reactivity, anxiety/depression, somatic complaints, and withdrawn behavior: findings that replicate those of Girard et al. [15]. These data suggest that breastfeeding’s protective effect may be especially pertinent to emotional problems, consistent with results from Meng et al. [20], who reported a lower prevalence of internalizing problems in children exclusively breastfed for 6 months and extended to 18 months. Nevertheless, as Turner et al. [21] emphasize, some of these effects may be mediated by contextual factors not always controlled, such as quality of the affective bond or maternal emotional state, emphasizing the need to interpret results within an ecological and multifactorial framework. In our study, by including parental emotional symptomatology and family structure (nuclear vs. other configurations) in adjusted models, we demonstrate that the association between any breastfeeding and child mental health persists even when considering these contextual characteristics.

Overall, our findings call for examining not only the duration of breastfeeding, but also the context in which it occurs. Emotional and behavioral problems in early childhood arise from complex interactions among biological, psychological, familial, and social factors [21]. During the early years, the child’s brain is in a period of high plasticity; thus, it is particularly sensitive to adverse experiences as well as protective factors [30]. Elements such as family environment, caregiver relationships, socioeconomic status, parenting practices, social support, and parental emotional well-being can significantly influence emotional and behavioral problems [21]. Our analysis aimed to account for some of this complexity by adjusting for a wide array of prenatal variables (gestational age, prenatal smoking), sociodemographic factors (maternal age, socioeconomic status, family structure), child nutritional characteristics, and parental emotional health; however, we acknowledge that other concurrent environmental influences remained unaccounted for. Accordingly, covariates were selected a priori based on previous literature identifying key determinants of child psychopathology and their availability in both cohorts. This strategy enables a more precise estimation of breastfeeding’s effect within a multifactorial, contextualized framework.

From a neurobiological point of view, breast milk provides essential long-chain polyunsaturated fatty acids that play a key role in myelination, synaptic connectivity, and overall development of the central nervous system [1]. However, the potential protective effect of breastfeeding on child mental health cannot be understood solely through nutritional mechanisms. Several studies propose that this effect may be partially mediated by socioenvironmental factors, such as home stimulation, quality of mother–child affective bond, and caregiver emotional state [21]. In this regard, breastfeeding can also be conceptualized as a relational practice that fosters affective attune and promotes secure attachment, which may contribute to more stable emotional development. This integrative framework enables viewing breastfeeding as part of a biopsychosocial matrix in which nutrition, attachment, and context dynamically interact.

Our results must be interpreted within the study’s limitations and strengths. Although early breastfeeding intensity may have contributed to the observed effects, this cannot be verified because feeding frequency and qualitative indicators of breastfeeding type were not collected. Additionally, as part of the sample was drawn from a longitudinal cohort, some attrition occurred over time, potentially introducing selection bias. However, analyses comparing participants retained at follow-up with those lost to follow-up revealed no significant differences in key sociodemographic or perinatal characteristics. Another limitation is that breastfeeding data include any breastfeeding (exclusive or mixed) without precise differentiation, an issue shared with much of the existing literature. Moreover, although maternal recall of breastfeeding duration is generally accurate, we acknowledge the possibility of inaccuracies due to retrospective reporting. Furthermore, since both breastfeeding information and behavioral outcomes were reported by parents, a possible bias arising from the use of the same informant for both measures cannot be entirely ruled out. Regarding sample origin, differences were noted between the EPINED and ECLIPSES cohorts, particularly in children’s sex distribution and presence of psychopathological symptoms. Although both cohorts are community-based, EPINED includes participants with clinical and subclinical symptoms of ADHD and ASD. To address this, sensitivity analyses stratified by cohort and models including the breastfeeding by cohort interaction were conducted, showing consistent associations across samples and no significant interaction effects. Even so, to mitigate potential bias, “cohort origin” was included as a covariate in the regression models, and we acknowledge that some residual underestimation may persist. Nevertheless, despite differences in study design, data collection for primary variables was performed uniformly, ensuring comparability.

Despite these limitations, one of this study’s major strengths is its inclusion of key variables relevant to emotional development, allowing for a rigorous assessment of breastfeeding’s potential role. In particular, adjusting for parental emotional health and family structure enhances the robustness of our findings by incorporating emotional and contextual dimensions closely linked to both child development and breastfeeding practices.

In conclusion, our results indicate that any breastfeeding, especially during the first 4 months of life, may act as a protective factor against the development of emotional and behavioral symptoms in early childhood. The protective association did not increase with longer durations, and in some specific outcomes, no advantages (or even slight elevations in risk) were observed when breastfeeding continued beyond 8 months. This suggests that the protective effect may be most relevant during the early postnatal period, when feeding intensity and maternal–infant contact could be greatest.

Rather than considering breastfeeding as an isolated determinant, it should be understood as part of a broader biopsychosocial network that includes parental emotional health, caregiving practices, early attachment, and family environment. These findings reinforce the importance of promoting breastfeeding as a strategy not only for nutritional benefits but also for supporting early emotional development and mental health. Further research is needed to disentangle the contributions of nutritional, relational, and contextual factors.

## Data Availability

No datasets were generated or analysed during the current study.
