# Correction to: Early breastfeeding as protective factor for preschool emotional and behavioral health

**DOI:** 10.1007/s00431-026-06838-1

**Published:** 2026-03-16

**Authors:** Susana Vargas-Pérez, Carmen Hernández-Martínez, Núria Voltas, Victoria Arija, Josefa Canals-Sans

**Affiliations:** 1 Research Group in Nutrition and Mental Health (NUTRISAM), Rovira I Virgili University, Tarragona, Spain; 2 Research Center for Behavioral Assessment (CRAMC), Rovira I Virgili University, Tarragona, Spain; 3 Pere Virgili Institute for Health Research (IISPV), Rovira I Virgili University, Reus, Spain

**Correction to: European Journal of Pediatrics (2025) 185:38**

10.1007/s00431-025-06666-9

In the article “Early breastfeeding as protective factor for preschool emotional and behavioral health” (Volume 185, Issue 1), an error occurred in the presentation of the authors’ names. The given names and family names were inverted, which may affect correct author identification and indexing in bibliographic databases.

The author names were incorrectly published as:

- Vargas-Pérez Susana
- Hernández-Martínez Carmen
- Voltas Núria
- Arija Victoria
- Canals-Sans Josefa

The correct presentation of the authors’ names is:

- Susana Vargas-Pérez
- Carmen Hernández-Martínez
- Núria Voltas
- Victoria Arija
- Josefa Canals-Sans

This correction ensures accurate attribution and indexing of the article in bibliographic databases.

The original article has been corrected.

